# CR6-interacting factor 1 deficiency reduces endothelial nitric oxide synthase activity by inhibiting biosynthesis of tetrahydrobiopterin

**DOI:** 10.1038/s41598-020-57673-9

**Published:** 2020-01-21

**Authors:** Ikjun Lee, Seonhee Kim, Harsha Nagar, Su-jeong Choi, Byeong Hwa Jeon, Shuyu Piao, Cuk-Seong Kim

**Affiliations:** Department of Physiology & Medical Science, School of Medicine, Chungnam National University, Daejeon, 301-747 Republic of Korea

**Keywords:** Aortic diseases, Carotid artery disease

## Abstract

Downregulation of CR6 interacting factor 1 (CRIF1) has been reported to induce mitochondrial dysfunction, resulting in reduced activity of endothelial nitric oxide synthase (eNOS) and NO production in endothelial cells. Tetrahydrobiopterin (BH4) is an important cofactor in regulating the balance between NO (eNOS coupling) and superoxide production (eNOS uncoupling). However, whether the decreased eNOS and NO production in CRIF1-deficient cells is associated with relative BH4 deficiency-induced eNOS uncoupling remains completely unknown. Our results showed that CRIF1 deficiency increased eNOS uncoupling and depleted levels of total biopterin and BH4 by reducing the enzymes of BH4 biosynthesis (GCH-1, PTS, SPR, and DHFR) *in vivo* and *vitro*, respectively. Supplementation of CRIF1-deficient cells with BH4 significantly increased the recovery of Akt and eNOS phosphorylation and NO synthesis. In addition, scavenging ROS with MitoTEMPO treatment replenished BH4 levels by elevating levels of GCH-1, PTS, and SPR, but with no effect on the level of DHFR. Downregulation of DHFR synthesis regulators p16 or p21 in CRIF1-deficient cells partially recovered the DHFR expression. In summary, CRIF1 deficiency inhibited BH4 biosynthesis and exacerbated eNOS uncoupling. This resulted in reduced NO production and increased oxidative stress, which contributes to endothelial dysfunction and is involved in the pathogenesis of cardiovascular diseases.

## Introduction

Endothelial dysfunction is the initial major process in the development of vascular diseases such as hypertension and atherosclerosis, as vascular endothelium plays an important role in maintaining vascular function and vascular homeostasis^1,2^. Nitric oxide (NO), produced by nitric oxide synthase (NOS), plays a critical role in regulating endothelial function and vascular tone^3^. In states of vascular disease, endothelial NOS (eNOS) bioactivity is reduced, and eNOS produces reactive oxygen species (ROS) instead of NO, which has been referred to as eNOS uncoupling^4^. A number of potential mechanisms are involved in eNOS uncoupling, including lack of NOS cofactor tetrahydrobiopterin (BH_4_), depletion of L-arginine, accumulation of methylarginines, and S-glutathionylation of eNOS^5–7^. Among these, the depletion of BH_4_ is likely the main cause of eNOS uncoupling, yet the detailed mechanism is not well understood.

It is well known that BH_4_ is synthesized in the cytoplasm of animal cells via *de novo* and recycling pathways. GTP cyclohydrolase I (GTPCH), as the first and rate-limiting enzyme in the de novo pathway, catalyzes the formation of dihydrobiopterin triphosphate from Guanosine triphosphate (GTP), which is then converted to 6-pyruvoyltetrahydropterin by 6-pyruvoyltetrhydropterin synthase (PTPS). Finally, 6-pyruvoyltetrahydropterin is reduced to BH4 by sepiapterin reductase (SR)^8^. In the recycling pathway, dihydropterin (BH2) can be reduced back to BH4 by the enzyme dihydrofolate reductase (DHFR), an enzyme-recycling oxidized BH4^9^. The oxidation of BH4 by ROS such as peroxynitrite results in the production of BH2, which inactivates eNOS function. This increases the possibility that BH4 deficiency resulting from excessive ROS production stimulates the initial stage in the development of vascular diseases^10,11^. Recent studies have suggested that BH4 supplementation improves vascular function in vascular diseases including coronary artery disease and hypertension^12,13^.

Furthermore, BH4 deficiency has been linked to reduced *de novo* synthesis under conditions of oxidative stress. Specifically, reduced production of BH4 was caused by downregulation of GTPCH1, PTPS, and SR or by reduced recycling from BH2 due to the downregulation of DHFR. Notably, GTPCH1 knockdown inhibited the serine 116 phosphorylation of eNOS and increased levels of uncoupled eNOS^14,15^. Moreover, DHFR deficiency also reduced BH4 levels, which resulted in eNOS uncoupling and mediated the development of hypertension^8,16^.

CR6 interacting factor 1 (CRIF1) is one of the largest mitoribosomal subunits and is essential for the synthesis and insertion of oxidative phosphorylation polypeptides (OXPHOS) in the mitochondrial membrane^17^. Therefore, a lack of CRIF1 is a major factor underlying misfolded mitochondrial OXPOS subunits. This deficiency leads to a production of excessive mitochondrial ROS in vascular endothelial cells which stimulates endothelial dysfunction^18^. Furthermore, CRIF1-deficiency-induced mitochondrial dysfunction stimulates impaired vascular function via the inactivation of eNOS and decreased NO production^19^. Recent evidence suggests that the mitochondrial ROS that has been linked to mitochondrial dysfunction also mediates the initiation of eNOS uncoupling^20,21^.

Mitochondrial dysfunction, including mechanisms of BH4 deficiency and eNOS uncoupling, is a known contributor to the development of vascular diseases. However, exactly how CRIF1-deficiency-induced mitochondrial dysfunction mediates the uncoupling of eNOS vascular endothelial cells remains unknown. In this study, we used siRNA-mediated knockdown of CRIF1 to explore the relative roles of CRIF1 deficiency and mitochondrial dysfunction in BH4 biosynthesis and recycling, as well as eNOS activity in vascular endothelial cells.

## Results

### CRIF1 deficiency induced eNOS uncoupling in HUVECs

**Figure 1:**
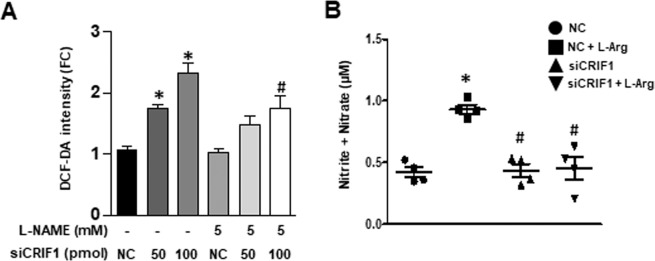
CRIF1 deficiency induced eNOS uncoupling in HUVECs. (**A**) Quantified DCF-DA fluorescence in control and CRIF1 siRNA treated cells with or without L-NAME (n = 3 per group; ^*^P < 0.05 vs control; ^#^P < 0.05 vs CRIF1 siRNA 100 pmol). (**B**) Nitrite and nitrate measurement in supernatant media from control and CRIF1 siRNA (100 pmol) treated cells with or without L-Arg (10 mM) (n > 3 per group; ^*^P < 0.05 vs control; ^#^P < 0.05 vs L-Arg).

CRIF1 knockdown disturbed the energy balance and mitochondrial function in endothelial cells and contributed to a higher concentration of ROS^22^. The increase in ROS may result from increased superoxide production or from uncoupled eNOS with reduced NO production. To confirm whether CRIF1-deficiency-induced ROS is derived from uncoupled eNOS generation, we incubated CRIF1-deficient cells with the NOS inhibitor L-NAME and observed a significant reduction in ROS levels at a siCRIF1 concentration of 100, but no effect at 50 pmol (Fig. 1A). These results suggest that eNOS may contribute to CRIF1 knockdown-induced ROS production. Coupled eNOS converts L-arginine to NO, whereas uncoupled eNOS produces superoxide, which may further reduce available NO. To determine the form of eNOS, we added 10 mM L-arginine 30 min before harvesting CRIF1 siRNA transfected HUVECs. Then, we tested NO production using a nitrate/nitrite colorimetric assay. As shown in Fig. 1B, NO generation was markedly increased in only the L-arginine treatment group; however, CRIF1 knockdown significantly inhibited L-arginine-induced NO production. These results suggest that CRIF1 deficiency limited the common substrate L-arginine to NO synthesis and resulted in eNOS uncoupling. These data suggested that eNOS uncoupling occurred in CRIF1-deficient endothelial cells.

### CRIF1 deficiency mediated BH4 biosynthesis diminution in HUVECs

**Figure 2:**
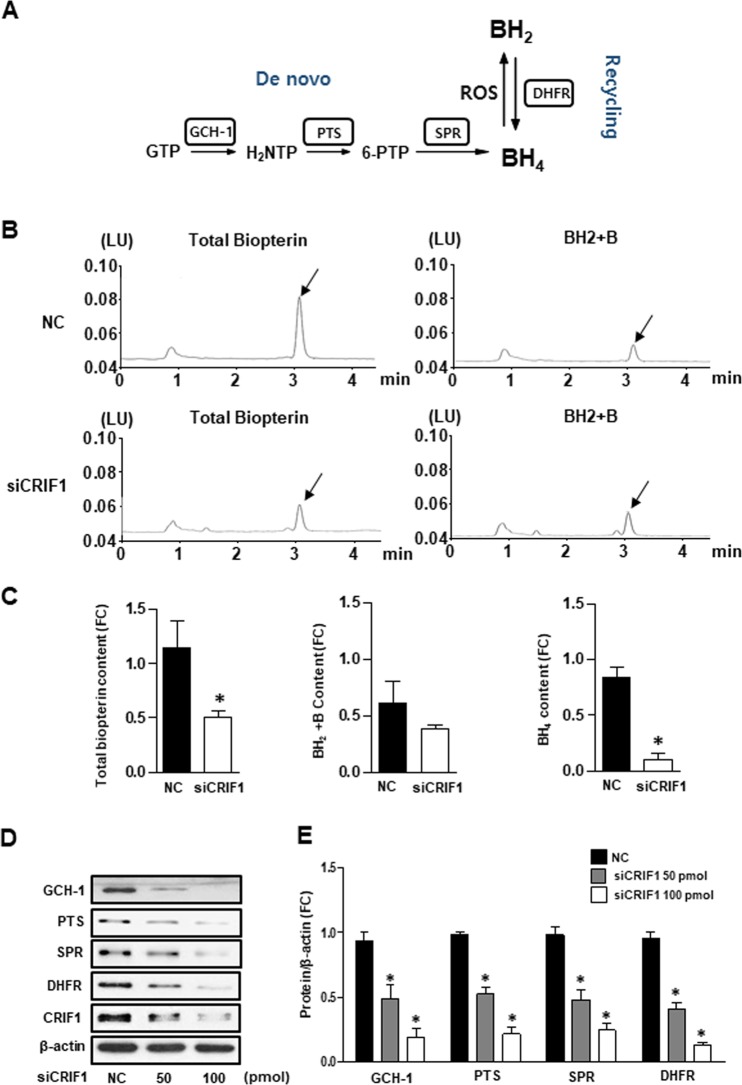
CRIF1 deficiency mediated BH4 biosynthesis diminution in HUVECs. (**A**) Schematic representation of the BH4 de novo pathway and recycling pathway. (**B**) Fluorescent signals of total biopterin and BH_2_ + B by fluorometric HPLC obtained from HUVECs with or without CRIF1 siRNA. Arrows indicated the signal of total biopterin and BH_2_ + B. (**C**) Quantification of total biopterin, BH_2_ + B and BH_4_ signal in endothelial cells with or without CRIF1 siRNA (n = 3 per group; ^*^P < 0.05 vs. control). (**D**, **E**) BH4 synthesis enzymes (GCH-1, PTS, SPR, and DHFR) expression in HUVECs with various amount of CRIF1 siRNA. Densitomeric analysis of each protein was shown in graph (n = 3 per group; ^*^P < 0.05, vs. control).

It is well known that eNOS uncoupling is linked to reduced BH4 bioavailability. BH4 is synthesized by de novo and recycling pathways from GTP and BH2, respectively (Fig. 2A). To determine the intracellular BH4 levels in CRFI1 deficient cells, we measured total biopterin (the sum of BH4, BH2, and biopterin) and BH2 + biopterin levels by using the HPLC method. As shown in Fig. 2B, the total biopterin levels were lower in CRIF1-deficient cells as compared with siCON cells, and there was no difference in the BH2 + biopterin levels. The BH4 concentration was calculated by subtracting BH2 + biopterin from total biopterin. Most notably, BH4 levels were significantly lower in CRIF1 knockdown cells (Fig. 2C). BH4 levels were determined by a balance of enzymatic de novo synthesis and recycling pathway. Next, we measured the levels of the BH4 synthesis enzymes GCH-1, PTS, SPR (*de novo* pathway), and DHFR (recycling pathway) in endothelial cells. As shown in Fig. 2D,E, the protein expression of these enzymes was decreased in CRIF1-deficient cells. These data suggest that CRIF1 deficiency reduced BH4 biosynthesis in HUVECs.

### BH4 is a key factor of CRIF1-deficiency-mediated eNOS uncoupling in HUVECs

**Figure 3:**
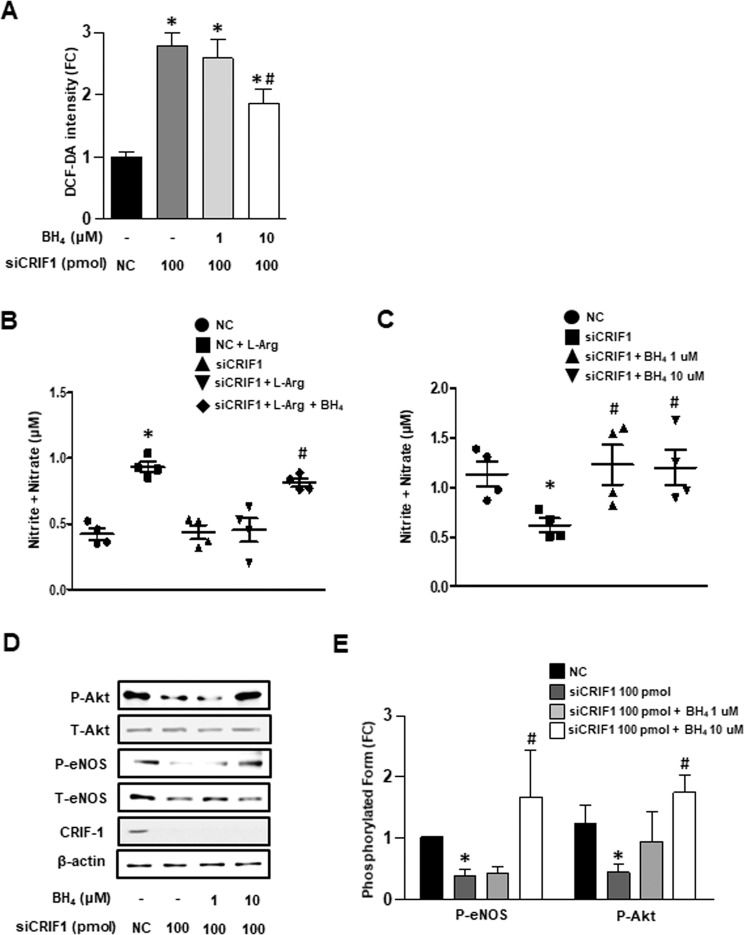
BH4 was key factor of CRIF1 deficiency-mediated eNOS uncoupling in HUVECs. (**A**) Quantified DCF-DA fluorescence of endothelial cells with different BH4 concentration and presence or absence of CRIF1 siRNA (n = 3 per group; ^*^P < 0.05 vs con; ^#^P < 0.05 vs CRIF1 siRNA 100 pmol). (**B**) Nitrite and nitrate measurement in supernatant media from control and CRIF1 siRNA treated HUVECs with or without BH4 (10 µM) and L-Arg (10 mM) (n = 3 per group; ^*^P < 0.05 vs control; ^#^P < 0.05 vs CRIF1 siRNA 100 pmol). (**C**) Nitrite and nitrate measurement in supernatant media from cells with different concentration of BH4 and with or without CRIF1 siRNA (n = 3 per group; *P < 0.05 vs. control; ^#^P < 0.05 vs CRIF1 siRNA 100 pmol). (**D**,**E**) Phosphorylation at serine 473 of Akt and phosphorylation at serine 1117 of eNOS in CRIF1-downregulated HUVECs with different amount of BH_4_. Measurement of band density was used to quantify protein expression (n = 3 per group; *P < 0.05 vs. control; ^#^P < 0.05 vs CRIF1 siRNA 100 pmol).

We showed that CRIF1 deficiency induced eNOS uncoupling and decreased BH4 biosynthesis in HUVECS. To further clarify whether eNOS uncoupling is associated with reduced BH4 levels, we examined the effect of BH4 on siCRIF1-triggered ROS generation and NO levels, respectively. As shown in Fig. 3A, treatment with 10 uM BH4 significantly decreased cellular ROS generation in CRIF1-deficient cells, suggesting that BH4 is a key factor of CRIF1-deletion-mediated ROS production. BH4 as an endogenous antioxidant could decrease ROS by its antioxidant effect or by increasing the formation of eNOS dimers. Our results show that BH4 augmentation not only recovered the ability of the substrate L-arginine for NO synthesis (Fig. 3B) but also improved NO generation in CRIF1-deficient cells (Fig. 3C). Therefore, the addition of BH4 may reduce CRIF1-deletion-induced ROS production mainly by uncoupled eNOS-triggered ROS. Moreover, given that Akt-dependent activation of eNOS contributes to NO production, we next investigated the expression of Akt/eNOS phosphorylation levels in the BH4-addition group. Figure 3D,E show that both Akt (serine 473) and eNOS (serine 1117) phosphorylation levels were elevated in CRIF1-deficient cells treated with the BH4 compared with CRIF1-deletion cells. Taken together, these data show that BH4 is a key factor in CRIF1-deficiency-mediated eNOS uncoupling in HUVECs.

### ROS mediated CRIF1-deficiency-induced eNOS uncoupling in HUVECs

**Figure 4:**
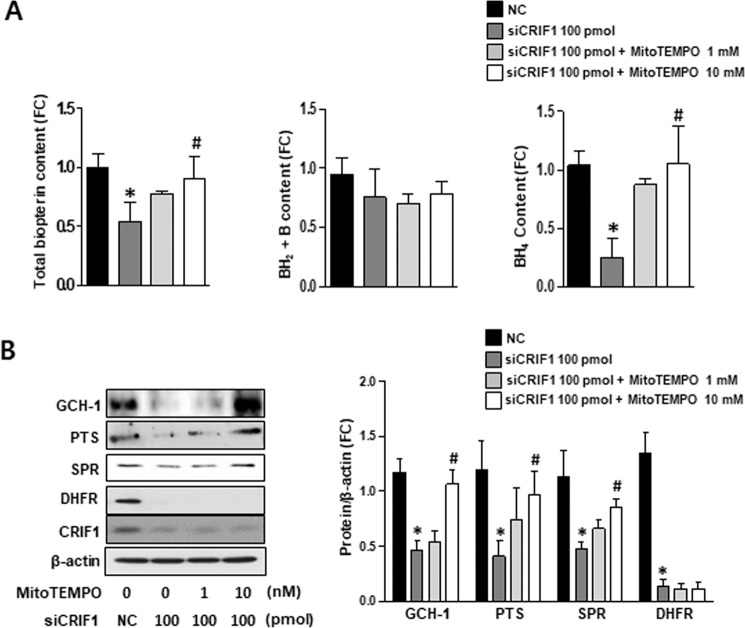
Mitochondrial ROS mediated CRIF1 deficiency-induced eNOS uncoupling in HUVECs. (**A**) Comparison of total biopterin, BH_2_ + B and BH4 content in control and cells with or without various amount of MitoTEMPO and CRIF1 siRNA. (**B**) BH4 synthesis enzymes (GCH-1, PTS, SPR, and DHFR) expression in control and CRIF1-deleted HUVECs pretreated with MitoTEMPO (1 mM or 10 mM). (**C**) Measurement of band density was used to analysis quantification of protein expression (n = 3 per group; *P < 0.05, vs. control; ^#^P < 0.05, vs CRIF1 siRNA 100 pmol).

Our previous studies showed that mitochondrial-specific O_2_^−^ scavenger Mito-TEMPO treatment rescued the NO levels that were reduced by CRIF1 deletion^22^. In this study, we revealed that BH4 supplements could also improve endothelial function by activating the Akt/eNOS/NO pathway. Therefore, we were curious whether CRIF1 knockdown-induced mitochondrial ROS leads to reduced BH4 synthesis, which is likely the major cause of eNOS uncoupling. We first eliminated mitochondrial ROS using mitochondria-specific ROS scavenger MitoTEMPO and measured the amount of total biopterin and BH2 + biopterin and calculated BH4 levels in both control and CRIF1-deficient groups with or without MitoTEMPO treatment. Our results showed that CRIF1-deficient cells treated with 10 mM MitoTEMPO had a significantly higher level of total biopterin and BH4 compared with CRIF1 siRNA transfected cells (Fig. 4A). Finally, the expression of enzymes linked with BH4 biosynthesis in endothelial cells was measured using western blot analysis. Figure 4B,C show that levels of GCH-1, PTS, and SPR proteins in CRIF1-deficient cells tended to recover with MitoTEMPO pretreatment. However, scavenging mitochondrial ROS did not result in a significant change in DHFR expression. Taken together, these finding indicate that the elevation of ROS was the major factor mediating eNOS uncoupling in CRIF1-deleted HUVECs. Furthermore, the elimination of ROS by antioxidants could induce eNOS recoupling. These mechanisms were related to BH4 bioavailability recovery via the *de novo* pathway.

### Cell cycle regulators p16 and p21 mediated DHFR synthesis in CRIF1-deficient HUVECs

**Figure 5:**
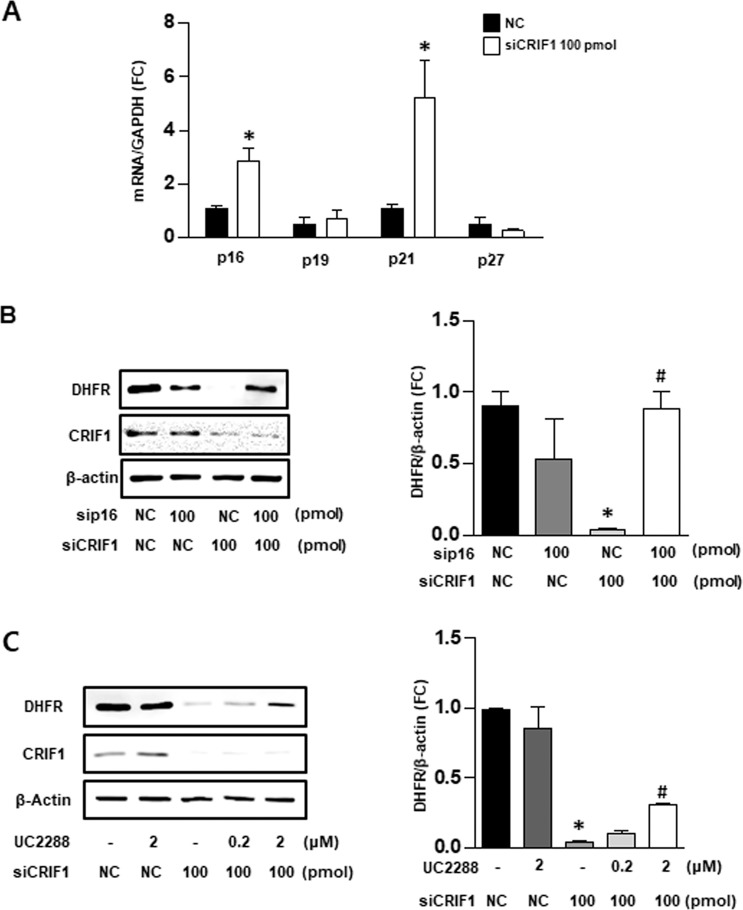
Cell cycle regulators p16 and p21 mediated DHFR synthesis in CRIF1-deficient HUVECs. (**A**) Quantification of mRNA levels of p16, p19, p21, p27 in control and CRIF1 siRNA treated HUVECs. (n = 3 per group; *P < 0.05 vs control) (**B**,**C**) DHFR protein expression in control and CRIF1 downregulated HUVECs with p16 siRNA or various amount of UC2288. Densitometric analysis for quantification of immunoblots of was shown (n = 3 per group; *P < 0.05, vs. control; ^#^P < 0.05 vs wCRIF1 siRNA 100 pmol).

To elucidate the mechanism by which the ROS scavenger failed to recover DHFR levels in CRIF1 siRNA-transfected cells, we examined the cell cycle-related genes involved in DHFR synthesis. DHFR synthesis initiates at the G1/S-phase boundary of the cell cycle, which is subject to biochemical regulators such as p16, p19, p21, and p27. In CRIF1-deficient cells, we found a significant increase in the mRNA levels of p16 and p21 compared to the siCON group (Fig. 5A). There was no difference in mRNA levels of p19 and p27 between groups. To determine whether the decreased level of DHFR resulted entirely from changes in the synthesis of regulators p16 and p21, we silenced p16 and p21 protein expression by p16 siRNA and p21 inhibitor (UC2288), respectively. We then observed DHFR protein expression in CRIF1-deficient cells. As shown in Fig. 5B,C, downregulation of either p16 or p21 partially prevented the decrease in DHFR protein levels after CRIF1 siRNA transfection. These findings indicate that the destruction of cell cycle regulators p16 and p21 plays a key role in the CRIF1-deficiency-induced DHFR reduction in endothelial cells.

### CRIF1 deficiency decreased BH4 bioavailability *in vivo*

**Figure 6:**
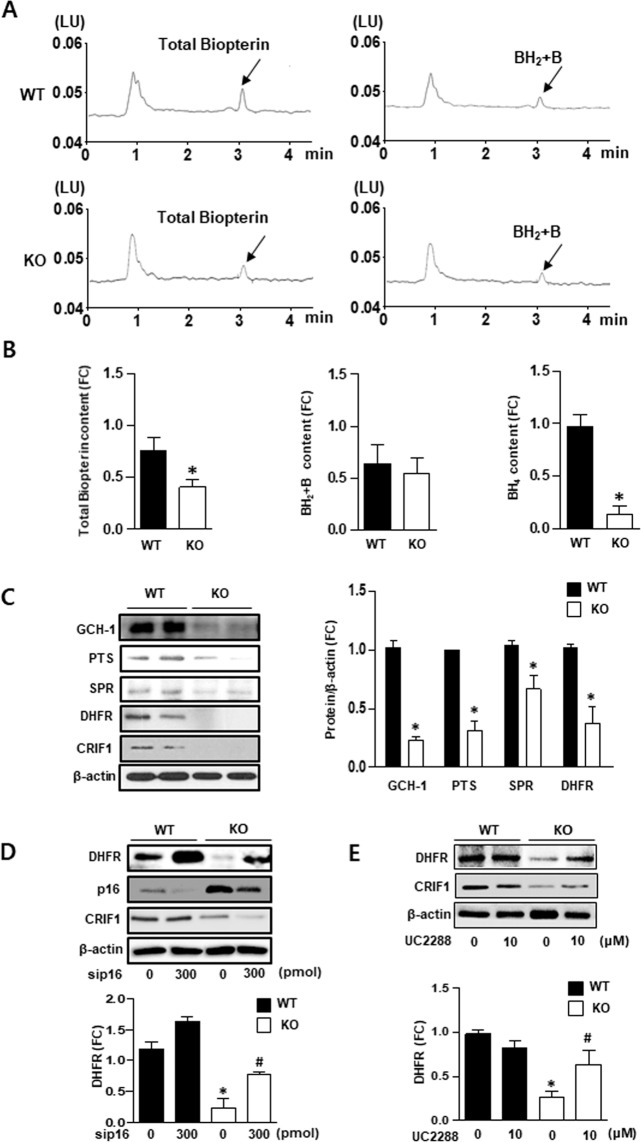
CRIF1 deficiency decreased BH4 bioavailability *in vivo*. (**A**,**B**) Fluorescent signals of total biopterin and BH_2_ + B in WT and CRIF1 KO mice lung endothelial cells. Arrows indicated the signal of total biopterin and BH_2_ + B. Quantification of total biopterin, BH_2_ + B and BH_4_ signal in endothelial cells were shown. (**C**) BH4 synthesis enzymes (GCH-1, PTS, SPR, and DHFR) expression in lung endothelial cells of WT and endothelial specific CRIF1 knockout mice. (**D**) DHFR protein expression in aorta of mice transfected with p16 siRNA. (**E**) DHFR protein expression in aorta of mice inhibited with p21 inhibitor (UC2288). Densitometric analysis of western blot was used for quantifying expression level of each protein (n = 3 per group; ^*^P < 0.05 vs WT).

To confirm BH4 bioavailability *in vivo*, we isolated lung endothelial cells from WT and endothelial-specific CRIF1 KO mice and then measured the amounts of total biopterin and BH2 + biopterin using HPLC analysis (Fig. 6A). The quantitative analysis of total biopterin, BH2 + biopterin, and BH4 levels is represented as a histogram. All levels were significantly attenuated in CRIF1 KO mice compared with WT mice (Fig. 6B). We next measured the levels of BH4 synthesis enzymes GCH-1, PTS, SPR (*de novo* pathway), and DHFR (recycling pathway) in isolated lung endothelial cells. The same results were observed *in vivo*, all enzyme levels were markedly diminished in CRIF1 KO mice compared with WT mice (Fig. 6C). To further explore the effect of p16 and p21 on DHFR synthesis *in vivo*, mice aortic rings were transfected with p16 siRNA or p21 inhibitor and then detected DHFR expression. Figure 6D,E shows that inhibition of p16 or p21 partially prevented the reduction of DHFR in CRIF1 KO mice, showing the same tendency observed in HUVECs. Taken together, these results suggest that CRIF1 deficiency decreased BH4 bioavailability *in vivo*.

## Discussion

**Figure 7:**
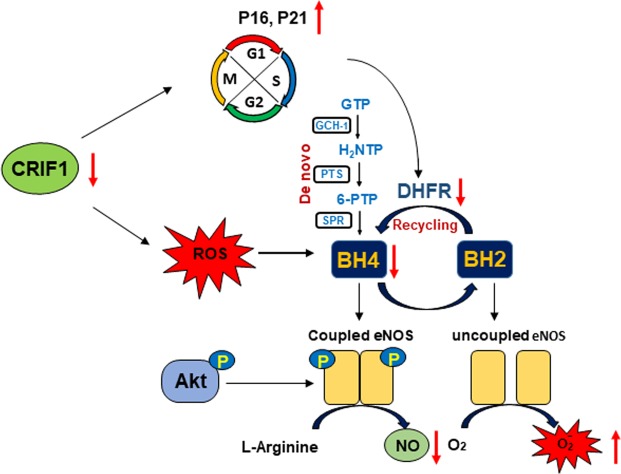
Schematic model depicting the regulation of CRIF1 on BH4 biosynthesis.

We have previously described that CRIF1-deficiency-induced mitochondrial dysfunction impaired vascular function by the Sirt1-eNOS pathway^22^. The present study highlights that decreased eNOS/NO production was caused by reduced BH4 levels. These results were determined by the inhibition of both *de novo* and recycling pathways, which resulted in eNOS uncoupling and ROS generation (Fig. 7). These findings show important insights into the role of CRIF1 in the homeostasis of vascular endothelial cells and the mechanisms underlying endothelial dysfunction and vascular diseases.

It is well known that mitochondria are a major source of ROS production and the overproduced ROS levels damage endothelial nitric oxide system and contributes to many cardiovascular diseases, such as atherosclerosis and hypertension^23–25^. The BH4 is an important cofactor in regulating ROS and NO production by shifting eNOS from an uncoupling to coupling form. Uncoupled eNOS in endothelial cells has been linked to decreased levels of BH4, which contributes to induced superoxide production instead of NO production in vascular diseases^26^. While we have previously described the effect of CRIF1 deficiency on ROS generation and eNOS inactivation in endothelial cells^22^, it is interesting to explore whether BH4-mediated ROS is associated with the vascular damage in CRIF1 downregulated cells. In this study, ROS production in CRIF1-deficient cells was reduced by treatment with the eNOS inhibitor L-NAME (Fig. 1A), suggesting that eNOS contributes to the increased ROS production in CRIF1-deficient vascular endothelial cells. In coupled eNOS, L-arginine can serve as a substrate for eNOS, producing NO and L-citrulline. However, in uncoupled eNOS, L-arginine may not increase the synthesis of NO production but does increase ROS production. To determine the form of eNOS, we specifically tested NO levels after L-arginine augmentation. Notably, there was no elevation in NO levels in CRIF1-deficient cells. These results confirmed our hypothesis that CRIF1 deficiency led to the reduction in NO levels partially through eNOS uncoupling in endothelial cells. Yang *et al*. showed that eNOS uncoupling was increased in aged vessels^27^. Yzydorczyk *et al*. indicated that eNOS uncoupling enhanced vascular superoxide, impaired endothelium-mediated vasodilatation, and decreased NO production in adult rats after transient neonatal oxygen exposure^28^. Although eNOS uncoupling has been implicated in the pathogenesis of vascular diseases, the underlying molecular mechanism of mitochondrial dysfunction has not been established until now.

BH4 is an essential cofactor for the aromatic amino acid hydroxylases and plays a vital role in the synthesis of NO. BH4 deficiency results in functionally uncoupled eNOS and therefore generates less NO and more oxygen free radicals. Given that CRIF1-deficient cells contributed to uncoupled eNOS, decreased NO generation, and increased ROS levels, we wonder whether there are some changes in BH4 levels. We found that both total biopterin and BH4 levels in CRIF1-knockout endothelial cells and in mice showed lowered expression compared with control groups (Fig. 2). In contrast to the increased BH2 + biopterin levels in different reduced BH4 cells^4,9^, CRIF1 deletion reduced total biopterin, but with no change in BH2 + biopterin levels, suggesting the decreased BH4 is not mainly induced by inhibition of BH2 recycling but by reduced BH4 synthesis (Fig. 2C). When insufficient cofactor BH4 was present, BH4 failed to stabilize the dimeric formation of eNOS and elevated the eNOS-dependent generation of ROS. On the other hand, the increased ROS further aggravated the oxidative situation by exacerbating eNOS uncoupling. Many studies have focused on the decreased ability of BH2 recycled back to BH4 via diminished DHFR expression^8,9,14^ Moreover, some studies investigated the *de novo* pathway of BH4 biosynthesis involving the detecting enzymes GCH-1^29,30^. To comprehensively understand the molecular mechanism of decreased BH4 in CRIF1-deficient cells, we specifically detected the enzyme expression in both *de novo* and recycling pathways. Our results showed that BH4 synthesis enzymes GCH-1, PTS, SPR (*de novo* pathway) and DHFR (recycling pathway) were significantly suppressed in CRIF1-deficient endothelial cells (Fig. 2D). These findings indicated that CRIF1-deficiency-induced mitochondrial dysfunction may disturb BH4 synthesis via *de novo* and recycling pathways, which have a direct effect on the inhibition of eNOS coupling and finally decreased NO production.

Since the defect in enzymes generation alters BH4 levels resulting in eNOS dysfunction during mitochondrial impairment, modulation BH4 levels may show an antioxidant effect and mediate increased expression in eNOS/NO activity. In addition, it has been documented that BH4 supplementation reduced endothelial damage and reserves vascular integrity^31^. So, to further confirm this hypothesis, we also supplemented BH4 in CRIF1-deficient cells, which resulted in a decrease in ROS levels (Fig. 3A) and a significant increase in NO production in HUVECS (Fig. 3C). These results mean that decreased NO production in the absence of CRIF1 is caused by reduced BH4 availability. Although the addition of BH4 led to a decrease in ROS production, it did not completely eradicate ROS production, which means that BH4 only partially inhibited ROS generation from uncoupled eNOS. The increased ROS in CRIF1-deficient cells not only originates from uncoupling eNOS but from mitochondrial dysfunction-induced oxidative stress. Although BH4 augmentation has effect on reducing ROS generation from uncoupled eNOS, the enhanced ROS derived from CRIF1 deficiency-induced mitochondrial dysfunction is hardly scavenged by BH4 treatment. In addition, since 10 uM BH4 totally recovered the Akt/eNOS phosphorylation levels and NO production, the concentration of BH4 used in this study is not the reason for partially abolishing ROS production. NO is synthesized from the amino acid L-arginine by the essential cofactor BH4. Several studies have reported that reduced levels of either BH4 or L-arginine resulted in eNOS uncoupling and a reduction in NO^32–34^. Therefore, other than the addition of BH4, supplementation of L-arginine may theoretically enhance NO production because of a decrease in eNOS uncoupling. However, some studies showed a beneficial effect of L-arginine, whereas others showed no beneficial effect of L-arginine in endothelial dysfunction models^35,36^. In the present study, we assessed the short-term effect of L-arginine supplementation on NO production and concluded that although the addition of arginine evidently elevated NO levels in control cells, there was no effect in CRIF1-deficient cells (Fig. 1A). The addition of BH4 rescued the CRIF1-deficiency-induced impaired NO generation, suggesting that the decreased BH4 in CRIF1-deficient endothelial cells with subsequent eNOS dysfunction may be a key determinant of impaired NO.

Delp *et al*. revealed that BH4 content (but not L-arginine) is reduced in vascular tissues in older rats due to age-related impairment of endothelium-dependent dilatation^35^. Furthermore, some studies showed that BH4 is susceptible to oxidation by reactive radicals, and increased ROS is associated with a deficiency in total biopterin and BH4 in mitochondria^37^. We have previously shown that CRIF1 deletion reduced the synthesis of OXPHOS and triggered severe ROS production in endothelial cells^17,18^. To further reveal whether CRIF1 knockdown induced ROS or whether CRIF1 itself impeded BH4 synthesis, we used the MitoTEMPO to remove mitochondrial ROS or free-radical scavenger N-acetyl-L-cysteine (NAC) to eliminate both cytosolic and mitochondrial ROS, and then detected changes in the levels of BH4 and NO. Surprisingly, removal of ROS using either MitoTEMPO or NAC elicited recovery of BH4/NO to normal levels by recovering the *de novo* pathway enzymes (GCH-1, PTS, SPR), but not the recycling pathway enzyme (DHFR) (Fig. 4 and Supplementary Figure 1). Because mitochondrial ROS in CRIF1 deficient cells act as a main trigger to induce ROS overproduction, therefore elimination of mitochondrial ROS showed the same results with NAC treatment. Although DHFR was not recovered, the levels of total biopterin and BH4 increased to control levels in endothelial cells with ROS scavenger treatment. GCH1 was reported to be a major determinant of BH4 bioavailability in vascular cells^38^. Furthermore, only when GCH1 was insufficient to meet the requirement of BH4 was DHFR recycled to total biopterin and BH4 content. This finding indicates that the recovered total biopterin and BH4 contents may be due to the increased GCH-1-mediated *de novo* pathway of BH4. This suggests that CRIF1-deficiency-induced ROS altered the *de novo* pathway of BH4 biosynthesis. Given that the reduced DHFR in CRIF1-deficient cells had no effect on ROS elimination, we hypothesized that CRIF1 deficiency may cause the inhibition of DHFR, but not ROS, through other pathways. Among the cell cycle-related genes involved in DHFR synthesis, only p16 and p21 showed a significant increase in CRIF1-deficient cells, and knockdown of either p16 or p21 recovered DHFR expression *in vitro* (Fig. 5B,C). Under our experimental conditions, it is difficult to generate double knockout mice that lack p16/p21 and CRIF1 to confirm the results *in vivo*. Although *ex vivo* aorta model has inherent limitations, it maintains the structure of the tissues with minimal alteration of natural condition and is widely used to assess endothelial function^39–41^. Therefore, we performed *ex vivo* downregulation of p16/p21 in thoracic aortas isolated from endothelial-specific CRIF1 knockout mice, which shows that p16/p21 inhibition also prevents the decreased-DHFR expression (Fig. 6D,E). Thus, the destruction of cell cycle regulators p16 and p21 plays a key role in CRIF1-deficiency-induced DHFR reduction in endothelial cells.

In summary, a considerable amount of research has established that BH4 is a vital cofactor of eNOS/NO production and ROS signaling in vascular physiology^30,42^. However, it is unclear whether endothelial cell BH4 deficiency contributes to impaired eNOS/NO function present in endothelial mitochondrial dysfunction. This study demonstrates that CRIF1-deficiency-induced oxidative stress inhibits the *de novo* BH4-synthesis pathway, and CRIF1 itself regulates recycling of the BH4-synthesis pathway and thereby leads to decreased NO production via eNOS uncoupling in the pathogenesis of cardiovascular diseases.

## Materials and Methods

### Chemicals and kits

N-acetylcysteine (NAC), L-NG-nitroarginine methyl ester (L-NAME), iodine, potassium iodide, phosphoric acid, ascorbic acid, L-arginine (L-Arg), and BH4 were purchased from Sigma-Aldrich (St. Louis, MO, USA). CM-H2DCFDA was obtained from Thermo Fisher Scientific (Waltham, MA, USA). The Nitric oxide assay kit and UC2288 were purchased from Abcam (Cambridge, UK).

### Cell culture

Human umbilical vein endothelial cells (HUVECs) were obtained from Clonetics (San Diego, CA, USA). The cell growth medium Endothelial Growth Medium-2 (EGM2) was purchased from Lonza (Walkersville, MD, USA). The cells were incubated at 37 °C with 5% CO_2_, as described in the manufacturer’s manual. HUVECs at passages 4 to 9 were used for experiments.

### Cell transfection and chemical treatment

For downregulation of the CRIF1 and p16 genes in HUVECs, short interfering RNA (siRNA) transfection was used. CRIF1 siRNA (sense: 5′-UGG AGG CCG AAG AAC GCG AAU GGUA-3′; antisense: 5′-UAC CAU UCG CGU UCU UCG GCC UCC A-3′), p16 siRNA (sense: 5′-CGC ACC GAA UAG UUA CGG U-3′; antisense: 5′-ACC GUA ACU AUU CGG UGC G-3′), and negative control siRNA were transfected with lipofectamine 2000 reagent purchased from Invitrogen (Carlsbad, CA, USA) according to the company’s protocol. HUVECs were plated in six-well plates and incubated with transfection mixture in 2 ml Opti-MEM for 4 h before being replaced with EGM2 culture medium. For CRIF1 knockdown, cells were incubated with CRIF1 siRNA for 48 h. After 24 h siCRIF1 transfection, p16 siRNA was transfected in the same manner as CRIF1 siRNA. NAC, L-NAME, and BH4 were used after 24 h siRNA transfection.

### Mouse studies

Generation of floxed CRIF1 (CRIF1flox/flox) mice was performed as previous studies. Tek-Cre mice were obtained from Jackson Laboratory (Bar Harbor, ME, USA). Genomic DNA was extracted from tail snips and used for genotype identification by PCR. 8–10 week-old WT and CRIF1 knockout mice were anesthetized (urethane, 1.3 g/kg, ip), and descending thoracic aortas were separated from the chest cavity and sectioned into 4 mm width rings. Aortic sections were incubated in EGM2 medium for 2 h and then treated with UC2288 (10 uM) for 24 h or transfected with scrambled siRNA mouse p16 siRNA using lipofectamine 2000 reagent. After 4 h, Opti-MEM used during transfection were changed to EGM2 and incubated for another 44 h. All samples were harvested with lysis buffer and used for western blotting. All animal experimental protocols conformed with the guidelines of the Institutional Animal Use and Care Committee and were approved by the institutional review board of Chungnam National University (CNUH-015-A0015). All animal studies were carried out in Chungnam National University Preclinical Research Center following their guidelines.

### Lung endothelial cells isolation

To extract the mouse endothelial cells, lungs of 8-week-old wild-type (WT) and CRIF1 endothelial knockout mice were isolated after anesthetization with urethane (1.3 g/kg, i.p.) and exposure of the thoracic cavity. Lungs were homogenized and incubated with collagenase buffer at 37 °C for 40 min with gentle shaking. Homogenates were filtered using a 100-μm filter and centrifuged for 5 min at 700 *g*. After centrifugation, samples were mixed with RBC lysis buffer and then washed in PBS. To separate endothelial cells (CD45 negative, CD31 positive) from homogenates, homogenates and anti-CD45 magnetic beads were incubated for 30 min at 4 °C. Samples were filtered using a MACS system. The remaining samples were mixed with anti-CD31 magnetic beads for 30 min at 4 °C and then filtered with the MACS. Samples binding with anti-CD31 beads were extracted and washed with PBS for use in further experiments.

### Antibodies and immunoblotting

CRIF1, GCH1, SPR, DHFR, and p16 antibodies were purchased from Santa Cruz Biotechnology (Santa Cruz, CA, USA). PTS antibodies were purchased from Thermo Fisher Scientific (Waltham, MA, USA). P-eNOS, T-eNOS, P-Akt, and T-Akt antibodies were acquired from Cell Signaling Technology (Beverly, MA, USA). Whole HUVECs and lung endothelial cell lysates from 8-week-old WT and CRIF1 knockout mice were collected for homogenization by RIPA buffer obtained from Cell Signaling. Then, 20 μg of the homogenates were used in the Western blotting experiments.

### Measurement of biopterin

Fluorometric HPLC analysis was performed to quantify biopterin as previously described, with slight modification. Briefly, HUVECs and mouse lung endothelial cells were homogenized with 180 μL of cell lysis buffer (50 mM pH 7.4 Tris-HCl, 1 mM DTT, 1 mM EDTA), and 0.1 μM neopterin was used as the internal standard. Then, 20 μL of protein-removing buffer (1.5 M HClO4 plus 2 M H3PO4) was added to the homogenates and centrifuged to collect the protein-free supernatant. Acid oxidation was used to quantify total biopterin, and 10 μL iodine solution (1% iodine in 2% KI solution) was added to 90 μL of the protein-free supernatants. To measure levels of BH2 and biopterin, alkaline oxidation was carried out by mixing 80 μL of the protein-free supernatants with 10 μL 1 M NaOH and iodine solution. All samples were incubated at room temperature for 1 h in the dark. After incubation, the alkaline oxidation samples underwent an acidifying step with 20 μL 1 M H3PO4. Before HPLC analysis, iodine was reduced through 5 μL of fresh ascorbic acid (20 mg/ml). A 120 SB-C18 column (Poroshell) was used to perform HPLC with a mobile phase of 5% MeOH (5:95 MeOH:water, v/v) at a rate of 1 ml/min. Fluorescence was measured using a 1290 series fluorescence detector (Agilent) at 350 nm excitation and 450 nm emission. The content of BH4 was determined by subtracting alkaline oxidation from acid oxidation. The content of biopterin was normalized with each protein concentration.

### Nitrite and nitrate measurement

Levels of NO were measured using a colorimetric assay kit purchased from Abcam. Specifically, the levels of nitrite and nitrate, NO’s metabolites, were quantified. After 24 h of transfection, the medium was replaced with phenol red and serum-free medium. Cells were then incubated with the new medium for 24 h. Next, the medium was collected, and a deproteinizing step was performed using a 10-kDa filter. A colorimetric assay was conducted following the manufacturer’s protocol. The amount of NO was quantified by subtracting the background, and values are expressed as total levels of nitrite and nitrate. To measure the amount of NO induced by L-Arg, cells underwent 30 min of pre-incubation in a phenol-free and FBS-free medium at the end of cell growth. The substrates were treated for 30 min at 37 °C in an incubator with 5% CO2. Finally, the supernatants were collected.

### Reactive oxygen species detection

ROS levels were determined by DCF-DA fluorescence and staining. Cells were transfected with siRNA for 48 h and washed with PBS. For DCF-DA fluorescent quantification, cells were dissociated from the cell culture plate by trypsinization. After another washing with PBS, the cells were incubated in growth medium including 10 μM DCF-DA for 30 min at 37 °C. After staining, the growth medium was removed by centrifugation at 5000 *g* for 5 min, and the cells were resuspended with PBS. To perform DCF-DA staining, cells were washed with PBS followed by transfection and then incubated with 10 μM DCF-DA for 30 min at 37 °C. Next, cells were washed with PBS, and the fluorescent signal was measured by a Promega GloMax Discover system (Madison, WI, USA) at 475 nm excitation and 500~550 nm emission.

### qPCR

Total RNA was obtained using TRIzol reagent and was reverse transcribed into cDNA using a Maxime RT-PCR Premix kit as per the manufacturer’s manual. qPCR reaction was performed using an SYBR Green kit with p16 (sense: 5′-GTG AGG GTT TTC GTG GTT CAC-3′, antisense: 5′-CTG GTC TTC TAG GAA GCG GCT-3′), p19 (sense: 5′-ACT GGA TTC CTG GAC ACC CTG-3′, antisense: 5′-AGC AGT GTG ACC CTC TTG AAC-3′), p21 (sense: 5′-GGA CAG CAG AGG AAG ACC ATG-3′, antisense: 5′-CGG CGT TTG GAG TGG TAG AA-3′), and p27 (sense: 5′-ACT CTG AGG ACA CGC ATT TGG-3′, antisense: 5′-TGG GGA ACC GTC TGA AAC ATT-3′). The primers for human glyceraldehyde 3-phosphate dehydrogenase (GAPDH, sense: 5′-TGA ACG GGA AGC TCA CTG G-3′, antisense: 5′-TCC ACC ACC CTG TTG CTG TA-3′) were used as the internal control.

### Statistical analysis

Statistical analysis was performed using SPSS (version 17.0) statistical software (SPSS, Inc., Chicago, IL, USA). Differences between two groups were evaluated using t-tests. One-way analysis of variance was performed for multiple comparisons, and Tukey’s test was carried out for *post hoc* analyses. Data are presented as mean ± SEM. A value of P ≤ 0.05 was considered to indicate statistical significance. All data are representative of at least three independent experiments.

## Supplementary information

Dataset 1.

## References

[1] Brandes RP (2014). Endothelial dysfunction and hypertension. Hypertension.

[2] Sitia S (2010). From endothelial dysfunction to atherosclerosis. Autoimmunity reviews.

[3] Kawashima S, Yokoyama M (2004). Dysfunction of endothelial nitric oxide synthase and atherosclerosis. Arteriosclerosis, thrombosis, and vascular biology.

[4] Landmesser U (2003). Oxidation of tetrahydrobiopterin leads to uncoupling of endothelial cell nitric oxide synthase in hypertension. The Journal of clinical investigation.

[5] Bendall JK (2005). Stoichiometric relationships between endothelial tetrahydrobiopterin, endothelial NO synthase (eNOS) activity, and eNOS coupling *in vivo*: insights from transgenic mice with endothelial-targeted GTP cyclohydrolase 1 and eNOS overexpression. Circulation research.

[6] Lin KY (2002). Impaired nitric oxide synthase pathway in diabetes mellitus: role of asymmetric dimethylarginine and dimethylarginine dimethylaminohydrolase. Circulation.

[7] Chen CA (2010). S-glutathionylation uncouples eNOS and regulates its cellular and vascular function. Nature.

[8] Crabtree MJ, Tatham AL, Hale AB, Alp NJ, Channon KM (2009). Critical role for tetrahydrobiopterin recycling by dihydrofolate reductase in regulation of endothelial nitric-oxide synthase coupling: relative importance of the de novo biopterin synthesis versus salvage pathways. The Journal of biological chemistry.

[9] Crabtree MJ, Hale AB, Channon KM (2011). Dihydrofolate reductase protects endothelial nitric oxide synthase from uncoupling in tetrahydrobiopterin deficiency. Free radical biology & medicine.

[10] Hink U (2001). Mechanisms underlying endothelial dysfunction in diabetes mellitus. Circulation research.

[11] Alp NJ (2003). Tetrahydrobiopterin-dependent preservation of nitric oxide-mediated endothelial function in diabetes by targeted transgenic GTP-cyclohydrolase I overexpression. The Journal of clinical investigation.

[12] Dikalova A, Aschner JL, Kaplowitz MR, Summar M, Fike CD (2016). Tetrahydrobiopterin oral therapy recouples eNOS and ameliorates chronic hypoxia-induced pulmonary hypertension in newborn pigs. American journal of physiology. Lung cellular and molecular physiology.

[13] Cunnington C (2012). Systemic and vascular oxidation limits the efficacy of oral tetrahydrobiopterin treatment in patients with coronary artery disease. Circulation.

[14] Sugiyama T, Levy BD, Michel T (2009). Tetrahydrobiopterin recycling, a key determinant of endothelial nitric-oxide synthase-dependent signaling pathways in cultured vascular endothelial cells. The Journal of biological chemistry.

[15] Wang S (2008). Acute inhibition of guanosine triphosphate cyclohydrolase 1 uncouples endothelial nitric oxide synthase and elevates blood pressure. Hypertension.

[16] Li Q (2019). Knockout of dihydrofolate reductase in mice induces hypertension and abdominal aortic aneurysm via mitochondrial dysfunction. Redox biology.

[17] Kim SJ (2012). CRIF1 is essential for the synthesis and insertion of oxidative phosphorylation polypeptides in the mammalian mitochondrial membrane. Cell metabolism.

[18] Nagar H (2014). CRIF1 deficiency induces p66shc-mediated oxidative stress and endothelial activation. PloS one.

[19] Herskovic A, Mauer E, Christos P, Nagar H (2017). Role of Postoperative Radiotherapy in Pathologic Stage IIIA (N2) Non-Small Cell Lung Cancer in a Prospective Nationwide Oncology Outcomes Database. Journal of thoracic oncology: official publication of the International Association for the Study of Lung Cancer.

[20] Lee DY (2013). Nox4 NADPH oxidase mediates peroxynitrite-dependent uncoupling of endothelial nitric-oxide synthase and fibronectin expression in response to angiotensin II: role of mitochondrial reactive oxygen species. The Journal of biological chemistry.

[21] Daiber A (2017). Crosstalk of mitochondria with NADPH oxidase via reactive oxygen and nitrogen species signalling and its role for vascular function. British journal of pharmacology.

[22] Nagar H (2017). CR6-Interacting Factor 1 Deficiency Impairs Vascular Function by Inhibiting the Sirt1-Endothelial Nitric Oxide Synthase Pathway. Antioxidants & redox signaling.

[23] Harrison D, Griendling KK, Landmesser U, Hornig B, Drexler H (2003). Role of oxidative stress in atherosclerosis. The American journal of cardiology.

[24] Alexander RW (1995). Theodore Cooper Memorial Lecture. Hypertension and the pathogenesis of atherosclerosis. Oxidative stress and the mediation of arterial inflammatory response: a new perspective. Hypertension.

[25] Bernal-Mizrachi C (2005). Vascular respiratory uncoupling increases blood pressure and atherosclerosis. Nature.

[26] Alkaitis MS, Crabtree MJ (2012). Recoupling the cardiac nitric oxide synthases: tetrahydrobiopterin synthesis and recycling. Current heart failure reports.

[27] Yang YM, Huang A, Kaley G, Sun D (2009). eNOS uncoupling and endothelial dysfunction in aged vessels. American journal of physiology. Heart and circulatory physiology.

[28] Yzydorczyk C (2013). Developmental programming of eNOS uncoupling and enhanced vascular oxidative stress in adult rats after transient neonatal oxygen exposure. Journal of cardiovascular pharmacology.

[29] Cai S (2002). GTP cyclohydrolase I gene transfer augments intracellular tetrahydrobiopterin in human endothelial cells: effects on nitric oxide synthase activity, protein levels and dimerisation. Cardiovascular research.

[30] Carnicer R (2012). Cardiomyocyte GTP cyclohydrolase 1 and tetrahydrobiopterin increase NOS1 activity and accelerate myocardial relaxation. Circulation research.

[31] Edgar KS, Galvin OM, Collins A, Katusic ZS, McDonald DM (2017). BH4-Mediated Enhancement of Endothelial Nitric Oxide Synthase Activity Reduces Hyperoxia-Induced Endothelial Damage and Preserves Vascular Integrity in the Neonate. Investigative ophthalmology & visual science.

[32] Govers R, Rabelink TJ (2001). Cellular regulation of endothelial nitric oxide synthase. American journal of physiology. Renal physiology.

[33] Katusic ZS (2001). Vascular endothelial dysfunction: does tetrahydrobiopterin play a role? American journal of physiology. Heart and circulatory physiology.

[34] Stroes E (1998). Origin of superoxide production by endothelial nitric oxide synthase. FEBS letters.

[35] Bevers LM (2006). Tetrahydrobiopterin, but not L-arginine, decreases NO synthase uncoupling in cells expressing high levels of endothelial NO synthase. Hypertension.

[36] Joshi MS (2007). Receptor-mediated activation of nitric oxide synthesis by arginine in endothelial cells. Proceedings of the National Academy of Sciences of the United States of America.

[37] Bailey J (2017). A novel role for endothelial tetrahydrobiopterin in mitochondrial redox balance. Free radical biology & medicine.

[38] Antoniades C (2008). GCH1 haplotype determines vascular and plasma biopterin availability in coronary artery disease effects on vascular superoxide production and endothelial function. Journal of the American College of Cardiology.

[39] Yayama K, Hiyoshi H, Imazu D, Okamoto H (2006). Angiotensin II stimulates endothelial NO synthase phosphorylation in thoracic aorta of mice with abdominal aortic banding via type 2 receptor. Hypertension.

[40] Kassan M (2017). MicroRNA-204 promotes vascular endoplasmic reticulum stress and endothelial dysfunction by targeting Sirtuin1. Scientific reports.

[41] Galley JC (2019). Antagonism of Forkhead Box Subclass O Transcription Factors Elicits Loss of Soluble Guanylyl Cyclase Expression. Molecular pharmacology.

[42] Schmidt TS, Alp NJ (2007). Mechanisms for the role of tetrahydrobiopterin in endothelial function and vascular disease. Clinical science.

