# Supplementary material for: CR6-interacting factor 1 deficiency reduces endothelial nitric oxide synthase activity by inhibiting biosynthesis of tetrahydrobiopterin
Source: Sci Rep. 2020 Jan 21;10:842. doi: 10.1038/s41598-020-57673-9 (PMC6972730; doi:10.1038/s41598-020-57673-9)
Supplement: Supplementary file 1 — Dataset 1. [file 41598_2020_57673_MOESM1_ESM.pdf]

CR6-interacting factor 1 deficiency reduces endothelial nitric oxide synthase activity by inhibiting biosynthesis of tetrahydrobiopterin

Ikjun Lee[1], Seonhee Kim[1], Harsha Nagar[1], Su-jeong Choi[1], Byeong Hwa Jeon[1], Shuyu Piao[1*], Cuk-Seong Kim[1*]

[1]Department of Physiology & Medical Science, School of Medicine, Chungnam National University, Daejeon 301-747, Republic of Korea

Supplementary Figure 1.

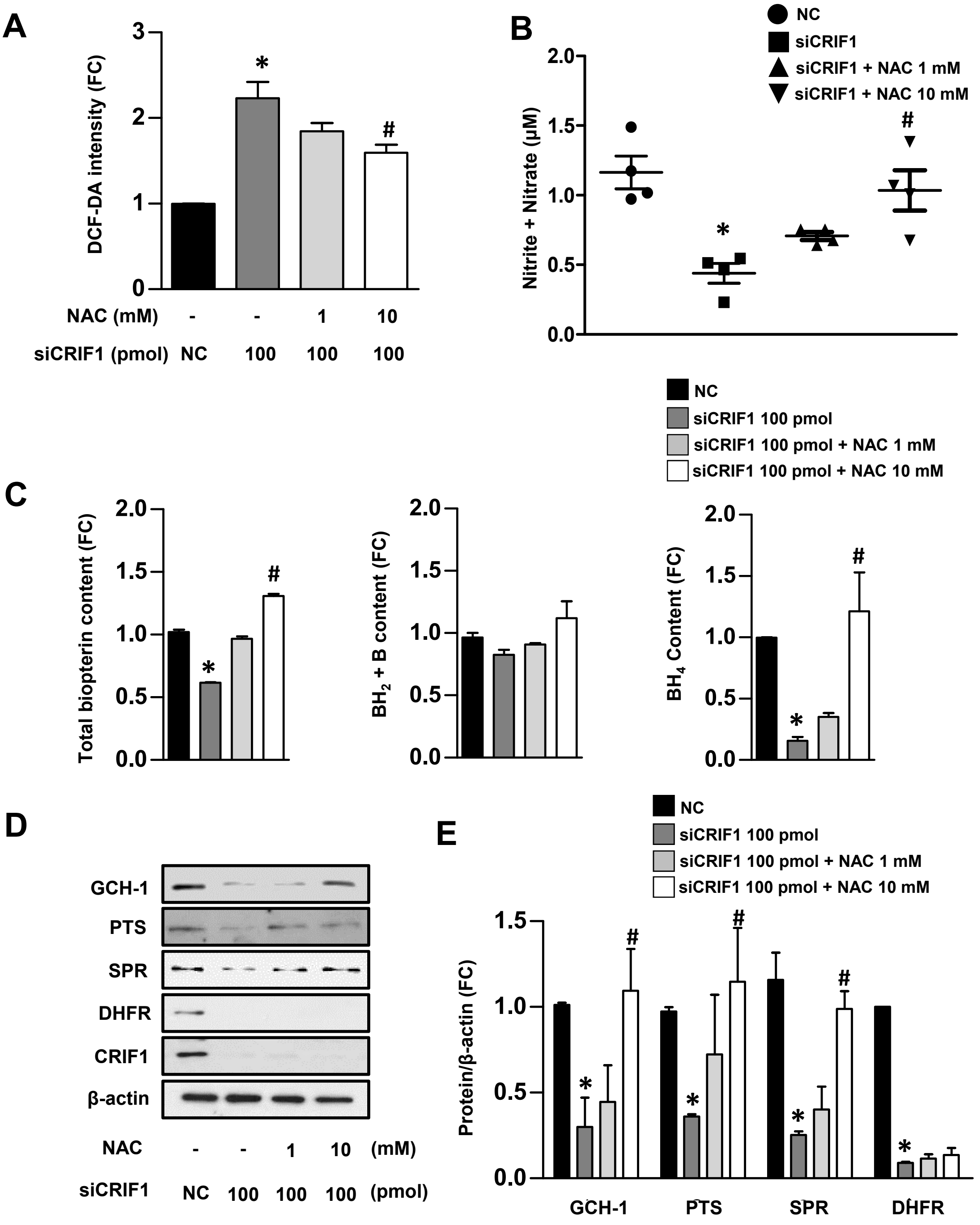

Supplementary Figure 1. ROS mediated CRIF1 deficiency-induced eNOS uncoupling in HUVECs.

(A) Measurement of fluorescence by DCF-DA staining in endothelial cells treated with different concentration of NAC and CRIF1 siRNA (B) Nitric oxide measured by sum of nitrite and nitrate in FBS free and phenol free supernatant media from CRIF1 deleted cells treated with various amount of NAC and CRIF1 siRNA. (D) Comparison of total biopterin, BH_2+B and BH4 content in control and cells with or without various amount of NAC and CRIF1 siRNA. (E, F) Expression of De novo and Recycling pathway in control and CRIF1 knockdown HUVECs with assorted amount of NAC. Measurement of band density was used to analysis quantification of protein expression (n=3 per group; *P<0.05, vs. control; #P<0.05, vs CRIF1 siRNA 100 pmol).
